# 544 Initial Experience Using a Biosynthetic Wound Matrix for Full-Thickness Wound Temporization

**DOI:** 10.1093/jbcr/iraf019.173

**Published:** 2025-04-01

**Authors:** Chinaemelum Akpunonu, Michael Young, Nidhi Aravapalli, Laura Pezzopane, Patrick Kennedy, Beth McGuire, Ariel Rodgers, Nicole Bernal, John Loftus

**Affiliations:** The Ohio State University, Wexner Medical Center; The Ohio State University, Wexner Medical Center; The Ohio State University; The Ohio State University, Wexner Medical Center; The Ohio State University College of Medicine; The Ohio State University; The Ohio State University, Wexner Medical Center; The Ohio State University, Wexner Medical Center; Ohio State Comprehensive Burn Center

## Abstract

**Introduction:**

Although immediate placement of an autograft is a fundamental principle of acute burn care, not all wounds or patients can undergo early skin grafting. Temporary dressings serve as a tool to seal the wound bed and act as a protective layer when grafting is not possible. Temporary dressings can also be used as a test graft or detect if remaining necrotic tissue is present. In less severe injuries, temporary dressings have been shown to facilitate re-epithelialization and pain control. Currently, frozen cadaveric tissue is an effective dressing for temporary coverage, but has limitations including cost, application time, small sizes, and potential for disease transmission. This is the first documented report of the use of a temporary biosynthetic bilayer wound matrix (BWM) comprised of an outer silicone layer with variable porosity and an inner tri-filament nylon matrix biocoated with collagen and aloe in lieu of frozen cadaveric tissue.

**Methods:**

A retrospective chart review was conducted for the first 3 patients treated in our center. Patients had acute full thickness wounds resulting from surgical excision of necrotic burn tissue and were not ready for immediate autografting. Following operative debridement, the BWM was secured in place and dressed with an absorbent pad, gauze roll, and compression wrap. Temporary biosynthetic bilayer wound matrix adherence, wound bed preparation, and complications are reported.

**Results:**

The average number of days until wound adherence was 3 and readiness for grafting was 5.6 days. TBSA range was 2%-3.9%. Wound locations included the thigh, bilateral lower extremities, and trunk. For the first 48 hours post-operatively there were no dressing changes and then the outer dressings were replaced daily until autografting. One of 3 patients developed a small hematoma, but it did not affect wound adherence. One patient had BWM placed as outpatient while the other two remained as inpatients.

Although a direct comparison was not made to cadaveric tissue, the BWM was easy to incorporate into practice and provided operative efficiencies. We found it faster to apply in the operative setting as it comes in large sheets (up to 2900 cm2), requires less staples, can be stored at room temperature, and does not have to thaw prior to application. We also noted the aftercare process was more efficient as it only required dry outer dressings and since the matrix is clear it facilitated rapid wound bed assessment.

**Conclusions:**

Our initial experience indicates that the temporary biosynthetic bilayer wound matrix was safe and effectively temporized the wound bed until grafting was possible.

**Applicability of Research to Practice:**

Temporary biosynthetic bilayer wound matrix should be considered as an alternative to cadaveric tissue for temporization of full-thickness wounds, as it provides OR efficiencies with no foreseen compromise to patient care.

**Funding for the Study:**

N/A

